# The Combined Beneficial Effects of Postbiotic Butyrate on Active Vitamin D3-Orchestrated Innate Immunity to *Salmonella* Colitis

**DOI:** 10.3390/biomedicines9101296

**Published:** 2021-09-22

**Authors:** Fu-Chen Huang, Shun-Chen Huang

**Affiliations:** 1 Department of Pediatrics, Kaohsiung Chang Gung Memorial Hospital and Chang Gung University College of Medicine, Kaohsiung 833, Taiwan; 2 Department of Anatomic Pathology, Kaohsiung Chang Gung Memorial Hospital and Chang Gung University College of Medicine, Kaohsiung 833, Taiwan; shuang@cgmh.org.tw

**Keywords:** butyrate, active vitamin D3, innate immunity, *Salmonella* colitis, vitamin D receptor

## Abstract

*Salmonella* spp. Remains a major public health problem globally. Biomedicine is the cornerstone of modern health care and could be a solution for antibiotic-resistant *Salmonellosis*. Although postbiotics seem to be an effective treatment in various clinical conditions, their clinical effects on *Salmonella* colitis have not been reported. Our previous report revealed that active vitamin D attenuates the severity of *Salmonella* colitis and invasiveness by reducing inflammation and enhancing the production of antimicrobial peptides. Therefore, we investigated the synergistic effects of butyrate, the most studied postbiotic, and active vitamin D on the severity of *Salmonella* colitis, invasiveness of *Salmonella*, and host immune responses, as well as its novel mechanisms, using in vitro and in vivo studies. We demonstrated that a combination of butyrate and active vitamin D (1 alpha, 25-dihydroxyvitamin D3) synergically reduced the severity of *Salmonella* colitis in C57BL/6 mice and reduced cecal inflammatory mIL-6, mIL-8, mTNF-α, and mIL-1β mRNA expression, but enhanced the antimicrobial peptide mhBD-3 mRNA, compared to a single treatment. Additionally, upregulated vitamin D receptor (VDR) plays a critical role in the synergistic effects. This suggests combined benefits of butyrate and active vitamin D on *Salmonella* colitis through VDR-mediated antibacterial and anti-inflammatory responses. The combined use of both supplements could be a potential biomedicine for infectious and autoimmune colitis.

## 1. Introduction

*Salmonella* spp. Are important Gram-negative pathogens of humans and animals, and which are known to cause a wide variety of diseases, ranging from mild diarrhea to severe systemic infections. Other complications include meningitis, osteomyelitis, sepsis, and toxic megacolon. Some types of *Salmonella*, such as *Salmonella enterica* serovar Typhimurium (ST) are becoming increasingly resistant to antibiotics. Antibiotic resistance may be associated with increased risk of hospitalization, development of a bloodstream infection, or treatment failure. Trends of increasing incidence of food-borne human infections caused by multi-drug-resistant strains of *S. typhimurium* have been noted globally [1], including in Taiwan [2]. Excess mortality was associated with antimicrobial drug-resistant *S. typhimurium* [3].

Probiotics are live microorganisms from the human gut that may confer benefits to the host for his health. Some of these beneficial effects are worked by postbiotics, which have recently been identified as a metabolic by-product generated by probiotics and to influence the host’s biological functions. Examples of postbiotics include short-chain fatty acids (SCFAs) such as acetate, butyrate, and propionate. The beneficial effects of SCFAs [4] on intestinal mucosa include (i) reducing production of inflammatory cytokines IL-1β, IL-6, and TNF-α by intestinal epithelial cells (IECs) through inhibition of the activation of NF-κB or histone deacetylase inhibitor (HDACi); (ii) enhancing the secretion of antimicrobial peptides (AMPs) from colon epithelial cells [5]; (iii) inhibiting the adhesion of pathogenic microorganisms to intestinal mucosa; (iv) selectively allowing the growth of probiotics over pathogenic microorganisms; and (v) stimulating epidermal growth factor receptor (EGFR), to increase mucin synthesis, leading to intestinal homeostasis. A study comparing probiotic and postbiotic activity in health and disease [6] showed that probiotics are not always beneficial for the host and can sometimes be detrimental in inflamed inflammatory bowel disease (IBD). By contrast, a potent postbiotic can downregulate ongoing inflammatory processes in IBD tissue. Therefore, postbiotics could be a safer alternative to the use of live probiotic organisms and might be the next wave of supplements used to nourish a healthy gut due to their discovered abilities; although the therapeutic benefit of these products in treating *Salmonella* colitis remains largely unknown. Among SCFAs, a wide spectrum of positive effects and multiple beneficial effects on human health exerted by butyrate has been well documented, with high potential for therapeutic use in human medicine.

Vitamin D deficiency has been correlated with increased rates of infection since the discovery that vitamin D induces AMP gene expression [7], while vitamin D3 supplementation reduces the odds of taking antibiotics by approximately 60% in patients with frequent respiratory tract infections [8]. A systemic review [9] provided evidence that 1,25D3 has the ability to ameliorate colitis by regulating innate intestinal immunity. Our recent study also observed that active vitamin D3 reduced *Salmonella* colitis by decreasing inflammation and bacterial translocation via the induction of killing and autophagic clearance of pathogenic organisms. The effect of adding unabsorbed carbohydrates, including dietary fibers and amylase- resistant starch to oral rehydration solution (ORS), on reducing acute diarrhea in adults and children [10] might be attributed to the SCFAs produced. SCFAs generated in the colon may help to relieve chronic diarrhea in children [11]. Propionate ameliorates DSS-induced colitis by improving intestinal barrier function and reducing inflammation and oxidative stress [12]. These findings suggest a combination of active vitamin D and SCFAs may treat *Salmonella* colitis by improving and/or modulating intestinal immunity.

Both vitamin D [13] and butyrate [14] have been shown to induce AMP cathelicidin expression in human colonic epithelial cells. However, the benefits of a combination of these two supplements on *Salmonella* colitis and invasiveness have not yet been reported. Therefore, we investigated the synergistic effects of butyrate, the most studied postbiotic, and active vitamin D on the severity of colitis, AMPs, and anti-inflammatory responses in *Salmonella* colitis mice.

## 2. Materials and Methods

### 2.1. Bacterial Strains

The ST wild-type strain SL1344 was grown in Luria–Bertani medium supplemented with 50 µg/mL streptomycin for 12 h at 37 °C and sub-cultured overnight in static cultures with minimal aeration. The bacteria were collected by centrifugation at 14,000× *g* for 5 min, washed with sterile phosphate-buffered saline (PBS), and resuspended in tissue culture medium without antibiotics at a density of 4 × 10^9^ CFU/mL.

### 2.2. Animal Experiments

All mice were generously provided by the National Laboratory Animal Center. At least forty-two 6- to 8-week-old female C57BL/6 mice were fed in specific-pathogen-free room in Kaohsiung Chang Gung Memorial Hospital animal center. Animal experiments were approved by the Kaohsiung Chang Gung Memorial Hospital Institutional Animal Care and Use Committee (Approval No. 2019092605, date: Dec 2020), according to the legal requirements. The *Salmonella* colitis model was conducted as previously described [15]. Briefly, mice were orally infected with 1 × 10^7^ CFU of SL1344 for 48 h after streptomycin treatment. For the experiments, the mice were housed in individually ventilated cages and transferred to a negative pressure room. Mice were divided into these groups: Control (open control), SL (SL1344 infected), VD (vitamin D3 and SL1344 infected), BT20 (Low dose butyrate and SL1344 infected), VD+BT20 (vitamin D3 plus Low dose butyrate and SL1344 infected), BT100 (High dose butyrate and SL1344 infected), and VD+BT100 (vitamin D3 plus high dose butyrate and SL1344 infected) (each group, n = 7). Before the colitis induction, mice were given vitamin D3 0.2 µg/25 g mice (VD group) or butyrate (BT group) or both vitamin D3 and butyrate (VD+BT) for 4 days via oral gavage. Other groups were fed 100 µL sterile water (Open control) or 100 µL PBS (SL group). After infection for 48 h, the mice were continually treated with vitamin D3 0.2 µg/25 g mice (VD group) or butyrate 20 or 100 mg/kg mice (BT group) or both (VD+BT group) for 7 days. Other groups were fed 100 µL sterile water (Open control) or 100 µL PBS (SL group). On day 14, submandibular bleeding was collected by using lancets. Animal body weight and diarrhea situation score were recorded during the experimental process. The diarrhea situation was scored as follows: 5 = normal stools and activity; 4 = a few wet and unformed stools; 3 = a number of wet and unformed stools with reduced mobility; 2 = severe and watery stool with weak and abnormal behavior; 1 = no activity upon prodding, convulsions, or death. We also calculated the spleen index as the assessment of immunity. Then, mice were sacrificed by CO2 narcosis and their intestinal tracts, spleen, and liver were removed, weighed, flushed with ice-cold PBS, and cut. Tissues were treated for further analysis.

### 2.3. Histological Colitis Scoring

Postmortem, the entire colon was removed and the colon length and weight were measured. Segments of the ileum, cecum, and colon were fixed and embedded in paraffin, according to the standard procedures. Alternatively, tissue samples were embedded in optimal cutting temperature compound (Sakura, Torrance, CA, USA), snap-frozen in liquid nitrogen, and stored at −80 °C. Part of the cecum were harvested and fixed in 10% formalin (pH7.4) and embedded in paraffin, according to standard protocols. Sections (5 μm) were stained with Hematoxylin and Eosin (H&E). Blinded histological scoring was performed using a validated scoring system by a trained pathologist. H&E sections were scored based on a study reported by Barthel et al. [16], with a combined score for submucosal edema (score, 0–3), polymorphonuclear granulocytes in the lamina propria (score, 0–4), number of goblet cells (score, 0–3), and epithelial integrity (score, 0–3) [16]. The combined pathological score for each tissue sample was determined as the sum of these scores; this ranged between 0 and 13 arbitrary units and covers the levels of inflammation in the intestine.

### 2.4. Quantitative Real-Time PCR Analysis of Cecum or Cultured Cells RNA

Samples of the cecum were obtained, immediately snap-frozen in liquid nitrogen, and stored at −80 °C. Total RNA was extracted from the cecum tissue or infected cultured cells, using TRI Reagent (Ambio #15596018) and Directzol RNA MiniPrep kit, according to the manufacturer’s instructions. The RNA was reverse-transcribed into cDNA using a PrimeScript™ RT reagent Kit (TaKaRaCat #RR037A) in a 20 µL reaction volume with a final concentration of 1 µg total RNA. Then, cDNA samples were subjected to quantitative real time PCR using a ABI 7500 Real-Time PCR System (Applied Biosystem) and FAST SYBR GREEN MASTER MIX, according to the manufacturer’s directions.

The primers (Genomics, New Taipei City, Taiwan) for the genes of interest and the internal reference were as follows: Chemokine (C-X-C motif) ligand 2 (CXCL2, an analog of human IL-8): forward, 5′-GCCCAGACAGAAGTCATAGCC-3′, reverse, 5′-GCTCCTCCTTTCCAGGTCAG-3′; Mouse beta defensin-3 (mBD-3, an analog of hBD-2): forward, 5′-GCATTGGCAACACTCGTCAGA-3′, reverse, 5′-CGGGATCTTGGTCTTCTCTA-3′; Mouse interleukin-1 beta (IL-1β): forward, 5′-AGCTTCCTTGTGCAAGTGTC-3′, reverse, 5′-TTGGGGTCCGTCAACTTCAA-3′; Mouse tumor necrosis factor-alpha (TNF-α): 5′-CTCCAGGCGGTGCCTATGTC-3′, reverse, 5′-CCATTTGGGAACTTCTCATCCCTTT-3′; Mouse interleukin-6 (IL-6): forward, 5′-GTTCCTCTCTGCAAGAGACTTC-3′, reverse, 5′-AGTCTCCTCTCCGGACTTGT-3′; Mouse Vitamin D Receptor (VDR): forward, 5′-ACCCTGGTGACTTTGACCG-3′, reverse, 5′-GGCAATCTCCATTGAAGGGG-3′; and Mouse beta-actin (β-actin): forward, 5′-TGT CGA GTC GCG TCC ACC-3′, reverse, 5′-TCG TCA TCC ATG GCG AAC TGG-3′.

We used an ABI 7500 Real-Time PCR System (Applied Biosystem) to set up the reactions. The reaction protocol was set as below. The denaturation step was set at 95 °C for 1 min, primer annealing step was set at 54 °C for 1 min, and the extension step was set at 72 °C for 2 min. These three steps were repeated for 25–35 cycles. The threshold cycle (Ct), which represents the duplication cycle number, was determined by the fluorescent value in the exponential phase of the amplification. ABI7500 software (SDS V2.3) was used to obtain raw fluorescence data (Rn and DRn) for analysis. The relative number of transcripts was normalized to the amount of β-actin transcript by subtracting the mean Ct value of the latter from the mean Ct value of the former for each experimental condition. The difference between the normalized Ct values of the infected cells and the control cells is a measure of the change in mRNA expression. Many aspects of the MIQE guidelines were taken into consideration for the methods and analysis [17].

### 2.5. Analysis of ST Loads in the Spleens and Livers

All the tissues aseptically removed from mice were weighed and recorded. To analyze the colonization of bacteria, the liver and spleen were homogenized in 4 °C cold PBS with 1% triton X-100 using a Potter homogenizer; using a micropestle to mince the tissue as much as possible. To determine the number of ST colonized, appropriate dilutions of the bacterial broth were cultured on MacConkey agar plates with 50 µg/mL streptomycin for 16 h at 37 °C under mild aeration. The minimal detectable values were 20 CFU/organ in the spleen and 100 CFU/organ in the liver.

### 2.6. Cell Culture and Infection

Caco-2 was purchased from the American Type Culture Collection (Manassas, VA, USA) and were cultured as described previously [18] or as recommended by the manufacturer.

### 2.7. Reagents

The butyrate was obtained from Sigma (St. Louis, MO, USA). The 1, 25-dihydroxyvitamin D3 (1,25D3) (Biomol Research Laboratories, Plymouth, PA, USA) was stored as a stock solution in pure ethanol at 22 °C in the dark. Standard laboratory reagents were from Sigma (St. Louis, MO, USA) or Fisher Scientific (Pittsburgh, PA, USA).

### 2.8. RNA Interference (RNAi) in Cultured Cells

RNAi experiments in cultured cells were performed as described previously [19,20,21]. Briefly, cultured Caco-2 cells were transfected with VDR siRNA according to the manufacturer’s protocol (SignalSilence^®^ Vitamin D3 Receptor siRNA I #12719) to knockdown VDR expression, as shown in our previous study [22]. After 48 to 72 h incubation in a 37 °C incubator, the transfected cells were infected by bacteria. Then the cells were lysed and RNA was extracted on ice for further analysis.

### 2.9. Statistical Analysis

All the above experiments were performed in triplicate with similar results. We made use of GraphPad Prism 8 software (GraphPad Software, San Diego, CA, USA) to perform the statistical analysis. For three or more nonparametric variables, we used Kruskal–Wallis one-way ANOVA to determine the variance. A *p*-value of <0.05 was considered statistically significant.

## 3. Results

### 3.1. A Combination of Butyrate and 1,25D3 Synergically Attenuates the Severity of Salmonella Colitis

**Figure 1:**
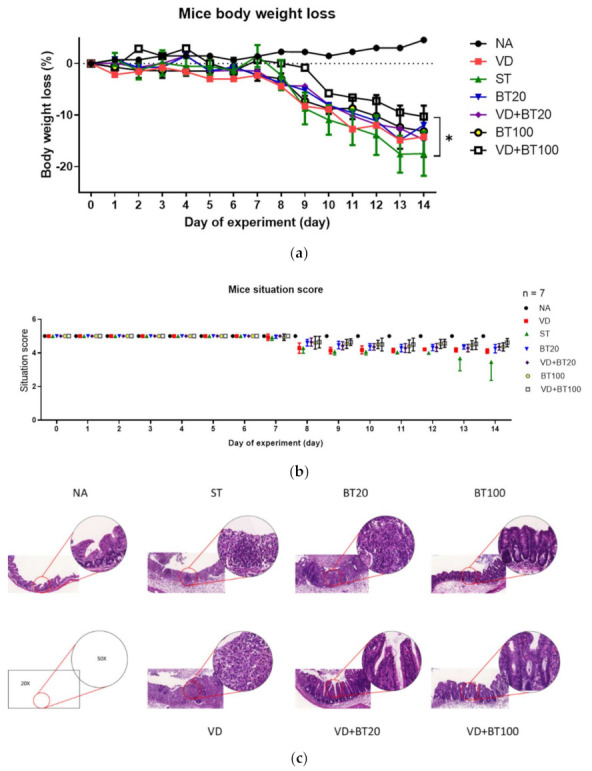

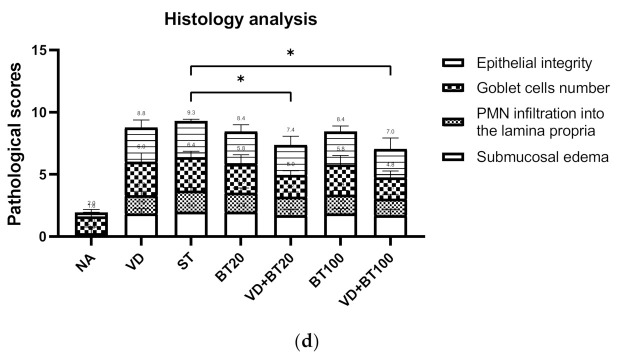
Combination of butyrate and active 1, 25 D3 attenuates the severity of *Salmonella* colitis in mice. First, 6–8-week-old female C57BL/6 mice (Charles River, Boston, MA, USA) were bred and housed under specific-pathogen-free conditions in the animal facility of the Center for Cellular and Biomolecular Research, Kaohsiung, Taiwan. Mice were fasted for 3 h and then orally administered 20 mg of streptomycin. Twenty-one hours after streptomycin treatment, mice were again fasted for 3 h and then orally infected with 10^8^ CFU of SL1344 (ST). Before infection, oral gavage with vehicle control (5% dimethyl sulfoxide), butyrate 20 or 100 mg/kg mice (BT20 or BT100) or 1α, 25(OH)_2_D_3_ (VD) treatment (1,25D3, 0.2 µg/25 g mouse) daily for 14 days was undertaken. Loss of body weight (**a**) and diarrhea situation scores (**b**) of mice were recoded daily. Cecum was resected and fixed in formaldehyde, and sections were stained with hematoxylin and eosin stain. (**c**) Representative histological images (×20 and ×50 magnification) of cecum from the different experimental groups. (**d**) The pathological scores (Supplementary Materials) for colitis in the cecum of mice. The data shown are means ± SEM (n = 7 mice/group). *, *p* < 0.05.

To study the effects of combined 1,25D3 and butyrate on the severity of *Salmonella* colitis, we examined the cecal pathology of infected WT mice untreated or treated by 1,25D3 and/or butyrate treatment. By histopathological analysis of the H&E-stained cecum sections, we observed obvious pathological changes of the cecum from the infected WT mice, as shown in Figure 1c and consistent with our previous study [15]. In contrast, decreases in the severity of *Salmonella* colitis were observed in the 1,25D3 or butyrate-treated ceca, but not significantly different from infection-only mice. Furthermore, a combination of both synergically attenuated the severity of *Salmonella* colitis, measured by the histological scoring system (Figure 1d).

In Figure 1, we can observe that a combination of high-dose butyrate and 1,25D3 significantly reduced the severity of *Salmonella* colitis, including body weight loss, situation of diarrhea, and pathologic scores in C57BL/6 mice; although low-dose butyrate had a beneficial effect, but not significantly.

### 3.2. Combination of 1,25D3 and Butyrate Attenuates Local Inflammation but Enhances Antimicrobial Peptide in the Cecum of Salmonella Colitis Mice

**Figure 2:**
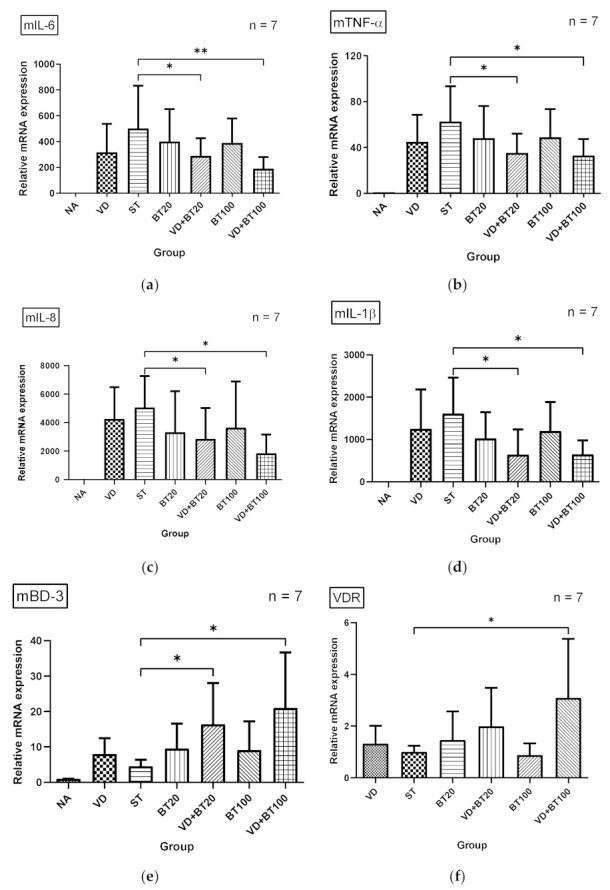
The immunomodulatory effects of 1,25D3 and butyrate on local cytokines and antimicrobial peptide in the cecum of *Salmonella* colitis mice. After *Salmonella* infection for 48 h, cecal tissues were obtained from the control, *Salmonella*-infected, and 1,25D3 and/or butyrate-treated infected mice. Total RNA was extracted from the cecal tissues. mIL-6 (**a**), mTNF-α (**b**), mIL-8 (**c**), mBD-3 (**d**), mIL-1β (**e**), and VDR (**f**) mRNA expressions were analyzed by quantitative RT-PCR. Values are measured as fold increase compared to the level of the control mice. The data shown are means ± the SEM (n = 7 mice/group). An asterisk indicates significant differences among groups, based on one-way ANOVA. *, *p* < 0.05, ** *p* < 0.01.

To investigate the effects of a combination of butyrate and 1,25D3 on the inflammatory and antimicrobial peptide (AMP) responses in *Salmonella*-infected mice, the gene expression of cytokines and AMP was analyzed by using quantitative real-time PCR (RT-PCR) in the cecal tissue of infected WT mice, untreated or treated with 1,25D3 and/or butyrate. Local gene expressions of cytokines and AMP (Figure 2) were significantly induced in the cecal tissue of *Salmonella* colitis mice. In contrast, cytokines, including IL-6, IL-8, TNF-α, and IL-1β, were synergically reduced in the cecal tissue of *Salmonella* colitis mice treated with a combination of 1,25D3 and butyrate, whereas AMP mBD-3 was synergically enhanced.

We observed that a combination of butyrate and 1,25D3 reduced cecal mIL-1β (643.5 ± 126.0 vs. 1606.0 ± 323.3, *p* < 0.05), mIL-6 (191.3 ± 34.1 vs. 493.7 ± 127.9, *p* < 0.05), and mTNF-α (33.12 ± 5.40 vs. 62.77 ± 11.60, *p* < 0.05) but increased mBD-3 mRNA (43.97 ± 9.73 vs. 62.77 ± 18.05, *p* < 0.05) expressions. Altogether, this suggests a synergistic effect of combined butyrate and active vitamin D on the severity of *Salmonella* colitis by enhancing antibacterial and anti-inflammatory responses.

### 3.3. Combination of Butyrate and 1,25D3 Exerted a Reduction of Bacterial Translocation in Salmonella-Infected Mice

**Figure 3:**
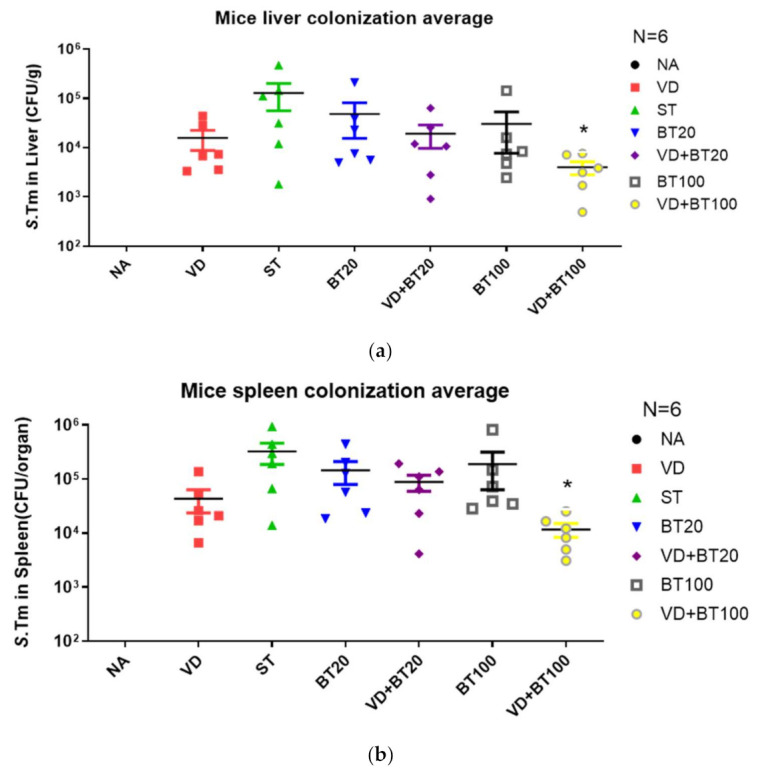
A combination of butyrate and 1,25D3 attenuates the systemic bacterial translocation of *Salmonella* colitis mice. Six to eight-week-old female C57BL/6 mice were infected with 10^8^ CPU of S. typhimurium (SL1344 strain) for 48 h, untreated or treated with 1, 25 D3 and/or butyrate daily to day 14. Numbers of bacteria were counted from the liver (**a**) and spleen (**b**) homogenates of control, *Salmonella*-infected, and 1,25D3, butyrate or combination-treated mice post-infection. The data shown means ± the SEM of the bacterial load in the liver and spleen (n = 6). *, *p* < 0.05.

Previously published data by Khailova et al. [23] and the authors [15] showed that 1,25D3 can reduce mortality and systemic bacterial translocation in experimental sepsis in weanling mice and *Salmonella* colitis mice. To determine the impact of a combination of butyrate with 1,25D3 treatment on bacterial invasion, liver and spleen tissues were obtained from *Salmonella* colitis mice treated with 1,25D3 and/or butyrate, homogenized and plated on LB plates. The CFU were determined. It was revealed that a combined butyrate and 1,25D3 treatment exerted a reduction of bacterial loads in the liver or spleen of *Salmonella*-infected mice (Figure 3).

We observed that a combination of butyrate and 1,25D3 reduced bacterial colonization (CFU/g tissue) in the liver (1.02 ± 0.20 × 10^2^ vs. 4.97 ± 0.66× 10^2^, *p* < 0.001) and spleen (1.50 ± 0.42× 10^2^ vs. 45.4 ± 3.56× 10^2^, *p* < 0.0001) compared to only SL1344 infection.

### 3.4. Combination of Butyrate and 1,25D3 Exerted Synergistic Effects on Vitamin D Receptor mRNA Expression in Salmonella-Infected Caco-2 Cells

In World J Gastroenterol [22], we demonstrated that active vitamin D enhances VDR mRNA and protein expression in *Salmonella*-infected intestinal epithelial cells (IECs). Additionally, we observed that a combination of butyrate and 1,25D3 enhanced cecal VDR mRNA expression, as shown in Figure 2. To investigate the regulatory effects of butyrate and active vitamin D on vitamin D receptor (VDR) mRNA expression in *Salmonella*-infected IECs, the cultured Caco-2 cells were either infected or not by SL1344 in the presence of butyrate, 1,25D3, or combination of both. Total RNA was prepared, reverse transcribed, and analyzed using real-time quantitative PCR.

**Figure 4:**
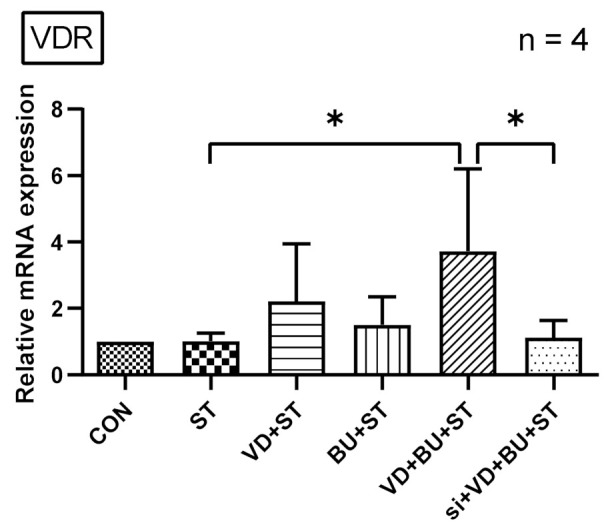
The synergistic effects of butyrate and active vitamin D on VDR mRNA expression in *Salmonella*-infected Caco-2 cells. Based on an in vivo study showing a combination of butyrate and 1,25D3 enhanced cecal VDR mRNA expression. Using an in vitro study, Caco-2 cells were not infected (CON) or infected by a constant concentration of S. typhimurium wild-typed strain SL1344 (ST), in the presence of butyrate (BU), 1,25D3 (VD), or a combination of both (VD + BU). Total RNA was extracted from Caco-2 cells and real-time quantitative PCR was performed to measure the amount of VDR mRNA. The results of the mRNA expression levels are depicted as means ± SEM of at least three independent experiments. * *p* < 0.05.

As shown in Figure 4, *Salmonella* infection induced VDR mRNA expression (normalized to GAPDH) in Caco-2 cells after one-hour infection, which was synergically enhanced using a combination of butyrate and 1,25D3. This suggests a synergistic effect of butyrate and active vitamin D on the VDR mRNA expression in *Salmonella*-infected IECs.

### 3.5. The Synergistic Effect of Butyrate on Vitamin D-Induced mRNA Expression Is Dependent on VDR

**Figure 5:**
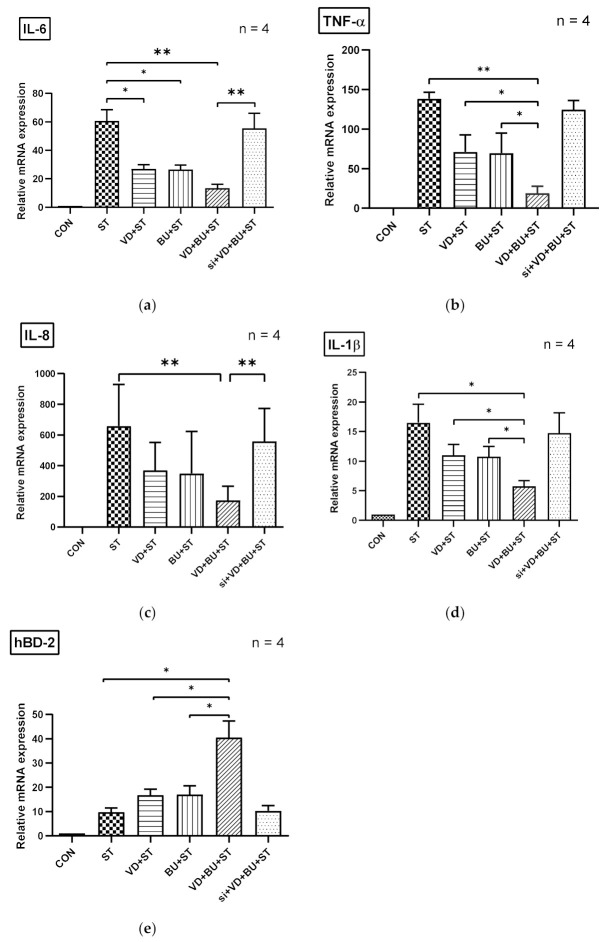
Involvement of VDR in synergistic effects of combined butyrate and active vitamin D3 on *Salmonella*-induced IL-6, IL-8, IL-1β, TNF-α, and hBD-2 mRNA expression in Caco-2 cells. Transfection of Caco-2 cells with control siRNA or VDR siRNA (si: siRNA to VDR) was carried out for 48 h. Transfected Caco-2 cells were infected by a constant concentration of S. typhimurium wild-type strain SL1344 (ST), in the presence of 1,25D3 (VD), butyrate (BU), or a combination of both (VD+BU); total RNA was extracted, reverse transcribed to cDNA, and RT-PCR analyses were performed to estimate amounts of IL-6 (**a**), IL-8 (**b**), TNF-α (**c**), IL-1β (**d**), and hBD-2 (**e**) transcript in *Salmonella*-infected cultured cells. Relative quantification based on internal reference gene (GAPDH transcript) to determine fold-differences in expression of the target genes, IL-6, IL-8, IL-1β, TNF-α, and hBD-2 mRNA were measured, and the fold increase over uninfected control cells (CON) is shown. Results are represented as mean ± SEM for at least three determinations from independent experiments. An asterisk indicates significant differences among groups, based on one-way ANOVA. *, *p* < 0.05, ** *p* < 0.01.

To further verify the role of VDR in the synergistic effects of butyrate on vitamin D-induced inflammatory responses and AMPs, we adapted a siRNA knock-down approach for VDR. Knock-down of VDR was confirmed by Western blot with specific siRNA in Caco-2 cells up to 48 hrs. siRNA-transfected Caco-2 cells were untreated (CON) or treated with vitamin D3 (VD), butyrate (BU) alone, or combination of butyrate and vitamin D3 (VD+BU) for 6 h. Following knockdown of VDR, we detected that the synergistic effect of butyrate on vitamin D3-induced mIL-6, mIL-8, mIL-1β, mTNF-α, and mBD-3 mRNA expression in Caco-2 cells was almost completely diminished in VDR-silenced cells (Figure 5), but not in control siRNA-silenced cells (data not shown). Therefore, specific suppression by siRNA targeting VDR diminished the synergistic mIL-6, mIL-8, mIL-1β, mTNF-α, and mBD-3 mRNA expression induced by a combination of butyrate and vitamin D3.

## 4. Discussion

We observed that a combination of butyrate and active vitamin D (1,25D3) synergically reduced the severity of *Salmonella* colitis and body weight loss in C57BL/6 mice by reducing cecal inflammatory mIL-6, mTNF-α, and mIL-1beta mRNA expression, but enhanced the antimicrobial peptide mhBD-3 mRNA, compared to a single treatment. This suggests a synergistic effect of butyrate and active vitamin D on the antibacterial and anti-inflammatory responses in *Salmonella* colitis. The combination use of both supplements could be a potential therapy for infectious and autoimmune colitis.

Among the SCFAs produced in the human intestine, butyrate has been shown to play an important role in the maintenance of colonic health. Many commensal bacteria produce butyrate in the intestine. Butyrate exerts multiple effects, such as the inhibition of colonic carcinogenesis, inflammation, and oxidative stress, as well as the improvement of colonic barrier function and the promotion of satiety. The addition of a diet with a amylase-resistant starch, a precursor of butyrate, to oral hydration solution reduced fecal fluid loss and led to more rapid recovery in adults with cholera [24] and children with acute diarrhea [25]. Butyrate reduced water and electrolyte secretion in rabbit colon [26]. Sodium butyrate shows great potential for replacing probiotics for ameliorating colitis via regulation of gut microbiota dysbiosis [27]. Moreover, butyrate protects IECs from *Campylobacter jejuni* invasion and translocation [28]. We demonstrated in this study the synergistic effect of active vitamin D3 and postbiotic butyrate on reducing inflammation, invasion, and translocation of *Salmonella* colitis in mice. This will open clinical interventions by using both bioactive agents to treat infectious diseases.

SCFAs could be important for communicating with intestinal epithelial and immune cells, thus affecting the expression of AMPs [29]. Actually, butyrate is a strong inducer of multiple β-defensin and cathelicidin genes in different porcine cell types [30]. Therefore, oral butyrate treatment in shigellosis induced endogenous cathelicidin CAP-18 in the colonic epithelium, promoted elimination of Shigella, and eased the symptoms of bacillary dysentery in rabbits [31]. Butyrate treatment augments antibacterial activity and bacterial clearance, leading to amelioration of inflammation, and these effects are likely due to the induction of endogenous synthesis of AMPs [32]. Two groups of investigators [33,34] showed that supplementation of phenylbutyrate reduced clinical illness in infections with *Shigellosis* in a rabbit model, with increased AMPs, cathelicidin, and defensins, and reduced levels of *Salmonella enterica* serovar Typhimurium (*S. Typhimurium*) gut colonization, invasion, and intestinal inflammation in mice. This interaction may help establish a mucosal barrier to prevent invasion of the intestinal epithelium by either mutualistic or pathogenic microorganisms. In a sepsis model in rats, induced by cecal ligation and puncture, injections of sodium butyrate prevented damage to multiple organs and reduced the lethality of severe sepsis [35].

The role of butyrate in attenuating pathogenic bacterial-caused hyperinflammatory responses was reported in a review [36]. Sodium butyrate attenuates inflammatory responses, neutrophil infiltration, and oxidative stress in the lungs, and protects against remote acute lung injury induced by severe burns [37]. These findings suggest diet and/or consumption of nutritional supplements may be used to improve and/or modulate immune function. In an ex vivo cultures of ileal and colonic mucosa from post-infectious irritable bowel syndrome patients, *Lactobacillus casei* DG (LC-DG) and its postbiotic significantly attenuated the inflammatory mucosal response [38], including IL-1α, IL-6, and IL-8 mRNA levels after LPS stimulation. SCFAs, of which butyrate was the most potent, affected the ability of leukocytes to migrate to the foci of inflammation and to destroy microbial pathogens [39]. Butyrate modulates the expression and release of IL-8, a chemokine recruiting neutrophils, by IEC in response to microbial infection, to modulate IEC-mediated neutrophil migration [40]. This can explain why a orally administered butyrate-releasing derivative reduced neutrophil recruitment and inflammation in murine colitis [41]. Clinically, oral butyrate is effective for inducing clinical improvement/remission in Crohn’s disease, which may exert its action through downregulating mucosal levels of NF-κB and IL-1β [42]. Sodium butyrate could promote porcine β-defensin 3 (pBD3) expression and inhibit IL-6 production upon toll-like receptor 2 activation and histone deacetylase inhibition in porcine kidney cells [43]. Our in vitro [22,44] and in vivo [15] studies observed that increased defensin expression in *Salmonella*-infected IECs and mice with *Salmonella* colitis using active vitamin D protected the host against infection, while downregulating proinflammatory responses (IL-6, IL-8, TNF-α, and IL-1β) protected the host from the detrimental effects of overwhelming inflammation, via vitamin D receptor (VDR). Therefore, in this study, we demonstrated that butyrate along with active vitamin D3 exerts synergistic effects on anti-inflammatory (IL-6, IL-8, TNF-α, and IL-1β), and enhances defensive (hBD-2), functions in *Salmonella* colitis. Butyrate is a metabolite of specific gut bacteria. Vitamin D has been shown to alter the gut microbiome composition and specifically increase butyrate-producing gut bacteria [45]. The synergistic effect of vitamin D with butyrate may be mediated through the gut microbiome.

On the other hand, administration of butyrate increased intestinal VDR expression and suppressed inflammation in a colitis model [46]. In a randomized controlled trial, they also found significant effects of oral phenylbutyrate and vitamin D3 adjunctive therapy in pulmonary tuberculosis [47]. The synergistic effect of tributyrin, a product of natural butyrate, and active 1,25D3 in Caco-2 cells was due to tributyrin-induced overexpression of VDR [48]. Treatment with tributyrin increased the binding of 1,25D3 to its receptor by 1.5-fold, without any change in receptor affinity. Furthermore, reversing bacterial-induced VDR dysfunction is key to autoimmune disease [49]. We observed that the synergistic effects of butyrate and active vitamin D3 in *Salmonella*-infected IECs depends on increased VDR expression. This may provide another mechanistic explanation and useful therapeutic approach for prevention and treatment of colitis, either infectious or autoimmune, using these two nutrients occurring naturally in human diet and enhancing VDR expression.

SCFAs levels in the intestine are much higher than in other tissues such as the liver or blood [50], suggesting that SCFA signaling, uptake, and/or metabolism mainly occur at the intestinal mucosa. In the human gastrointestinal tract, the highest SCFA concentration is found in the colon. Among SCFAs, butyrate, in particular, has cell type-specific effects in the intestinal epithelia and may be linked to local concentrations, where differentiated IECs in the villus are exposed to higher concentrations compared to the stem cells in the crypt [51]. Depending on the SCFA concentration, the effects of butyrate on immune responses can be higher, independent of receptors [52]. According to our study, a higher concentration of butyrate may have more synergistic effects on active vitamin D-orchestrated innate immunity against intestinal *Salmonella* infection than a lower concentration. However, these findings generate new research questions for patient therapy: what is the best concentration to induce gut innate immunity and immune tolerance?

### Limitations

The mechanisms of regulation of VDR expression and activity are complex [53], including (1) extracellular signals (ligands); 1,25(OH)_2_D3 increases VDR-mRNA production, stabilizes VDR-mRNA, and protects the VDR against degradation, and pathogen-induced activation of toll-like-receptors results in upregulation of the VDR; (2) intracellular signaling pathways (different intracellular signaling pathways, even in different cell types, might cooperate to regulate the expression of VDR); (3) transcription regulation (extracellular signals, so-called nutrigenomics [54], may enhance VDR gene transcription); (4) post-transcriptional modifications (disease-induced posttranslational modifications lead to a dysfunctional VDR), (5) co-regulators of VDR (VDR bound to DNA interacts with various co-activators or co-repressors to either induce or suppress gene transcription [55]); and (6) nuclear transportation. Thus, this deserves extensive and fully designed future studies to explore the true mechanisms by which butyrate enhances vitamin D-mediated VDR expression and activity; although, we have demonstrated the involvement of VDR in the combined benefits of postbiotic butyrate on vitamin D-orchestrated innate immunity in *Salmonella* colitis. Moreover, clinical trials are needed to determine the exact dosage that is most effective.

## 5. Conclusions

These findings not only demonstrate how butyrate and vitamin D can strengthen the human body’s innate immunity against invasion of *Salmonella* infection, but also reduce the waste of unnecessary antibiotics and hospitalization, increase social productivity, and reduce national economic losses. Due to their ability to decrease inflammation and defend against the invasion of microorganisms, postbiotics and vitamin D might be the next wave of supplements used to foster better gut health. Globally, this will inevitably make a great contribution to *Salmonella* infection control, and the same theory could be applied to various pathogens; while this therapeutic strategy can be extended to the study of other infections.

## Data Availability

The data presented in this study are available on request from the corresponding author.

## Supplementary Materials

The following are available online at https://www.mdpi.com/article/10.3390/biomedicines9101296/s1, Table S1: The pathological scores with mean and standard error for each group.
