# Supplementary material for: The Combined Beneficial Effects of Postbiotic Butyrate on Active Vitamin D3-Orchestrated Innate Immunity to Salmonella Colitis
Source: Biomedicines. 2021 Sep 22;9(10):1296. doi: 10.3390/biomedicines9101296 (PMC8533643; doi:10.3390/biomedicines9101296)
Supplement: Supplementary file 1 [file biomedicines-09-01296-s001.zip › biomedicines-1364321-supplementary.pdf]

Table S1: The pathological scores with mean and standard error for each group.

	Submucosal edema	PMN infiltration	Goblet cells number	Epithelial integrity	Total
NA	0.28 ± 0.18	0.00 ± 0.00	1.35 ± 0.12	0.31 ± 0.08	1.95 ± 0.25
VD	1.86 ± 0.14	1.48 ± 0.12	2.70 ± 0.25	2.75 ± 0.22	8.79 ± 0.70
ST	2.00 ± 0.00	1.70 ± 0.09	2.69 ± 0.17	2.90 ± 0.05	9.29 ± 0.31
BT20	2.00 ± 0.00	1.53 ± 0.14	2.31 ± 0.28	2.60 ± 0.20	8.45 ± 0.53
VD+BT20	1.71 ± 0.18	1.48 ± 0.15	1.76 ± 0.13	2.42 ± 0.26	7.38 ± 0.63
BT100	1.86 ± 0.14	1.51 ± 0.13	2.44 ± 0.27	2.64 ± 0.17	8.45 ± 0.54
VD+BT100	1.71 ± 0.18	1.34 ± 0.14	1.70 ± 0.19	2.29 ± 0.33	7.05± 0.80

*NA (Open control), ST (SL1344 infected), VD (vitamin D3 and SL1344 infected), BT20 (Low dose butyrate and SL1344 infected), VD+BT20 (vitamin D3 plus Low dose butyrate and SL1344 infected), BT100 (High dose butyrate and SL1344 infected), VD+BT100 (vitamin D3 plus high dose butyrate and SL1344 infected) (each group, n = 7)
